# Human activated protein C variants in a rat model of arterial thrombosis

**DOI:** 10.1186/1477-9560-6-16

**Published:** 2008-10-29

**Authors:** Karl Malm, Björn Arnljots, Björn Dahlbäck

**Affiliations:** 1 Department of Clinical Sciences, Division of Reconstructive Surgery, Lund University, University Hospital, SE-20502 Malmö, Sweden; 2 Department of Laboratory Medicine, Division of Clinical Chemistry, Lund University, University Hospital, SE-20502 Malmö, Sweden

## Abstract

**Background:**

Activated protein C (APC) inhibits coagulation by degrading activated factor V (FVa) and factor VIII (FVIIIa), protein S (PS) functioning as a cofactor to APC.

**Methods:**

By mutagenesis of the vitamin K-dependent Gla domain of APC, we have recently created an APC variant having enhanced anticoagulant activity due to increased affinity for negatively charged phospholipid membranes. In the present study, the potential antithrombotic effects of this APC variant, and of a variant APC that is additionally mutated in the serine protease domain, have been evaluated in a blind randomized study in a rat model of arterial thrombosis. In this model, we have previously found the combination of bovine APC and PS to be highly antithrombotic. Four treatment groups each containing 10 rats were, in a blind random fashion, given intravenous bolus injections of wild-type or mutant variants of APC (0.8 mg/kg) together with human PS (0.6 mg/kg) or human PS (0.6 mg/kg) alone. A control group with 20 animals where given vehicle only.

**Results:**

A trend to increased patency rates was noted in a group receiving one of the APC variants, but it did not reach statistical significance.

**Conclusion:**

In conclusion, administration of human APC variants having enhanced anticoagulant efficacy together with human PS in a rat model of arterial thrombosis did not give an efficient antithrombotic effect. The lack of effect may be due to species-specific differences between the human protein C system and the rat hemostatic system.

## Background

Activated protein C (APC) is an endogenous anticoagulant, which is crucially important for the regulation of blood coagulation [1-6]. APC is generated by limited proteolysis of its zymogen protein C by thrombin bound to its cofactor thrombomodulin on the surface of intact endothelium. APC effectively down-regulates the coagulation processes by selectively degrading the activated coagulation cofactors factor V (FVa) and factor VIII (FVIIIa). Protein S (PS) is a cofactor to APC in the regulation of FVa and FVIIIa. The importance of protein S as a cofactor in the anticoagulant protein C system is illustrated by the increased risk of venous thrombosis in individuals with a hereditary deficiency of the protein [7,8]. The molecular mechanisms involved in the enhancement by PS in the APC-reactions are incompletely understood. PS has been suggested to increase the affinity of APC to phospholipid membranes [9] but also to alter the orientation of the active site of APC [10].

Because of its powerful anticoagulant features and selective action, APC is interesting to evaluate for therapeutic intervention and APC has been shown to have antithrombotic features in different animal thrombosis models. Thus, human APC (hAPC) is highly effective in non-human primates [11-15]. Bovine APC (bAPC) is also highly antithrombotic in arterial thrombosis models in rabbits and rats, but only when co-administered with bovine protein S (bPS), demonstrating the importance of PS as a cofactor to bAPC and the species specificity in the APC-PS interaction [16-19]. The results using hAPC in rat models are more divergent. Earlier work showed antithrombotic effects of even low doses of hAPC in different models of thrombosis in the rat [20-24]. However, we have been unable to demonstrate any antithrombotic effects of hAPC in an arterial thrombosis model in rats[25]. This model is dependent on both primary hemostasis and on activation of blood coagulation, as indicated by the antithrombotic effects of active-site inhibited FVIIa, heparin and the combination of bovine APC and bovine protein S[19,26,27].

Recently, we have created recombinant human APC (hAPC) variants that have enhanced anticoagulant effects. Selective mutagenesis of the phospholipid-binding Gla domain generated the mutant QGNSEDY-hAPC, which has increased affinity for negatively charged phospholipids membranes and enhanced anticoagulant activity[28,29]. The protease domain has also been subjected to mutagenesis aiming at improving the anticoagulant activity of APC. By replacing the so-called 148 loop in hAPC with the shorter bovine loop (the variant denoted hAPC: B148), the catalytic and anticoagulant activity was found to be enhanced [30]. By combining the mutations above, a new mutant QGNSEDY-hAPC: B148 was constructed that due to the combination of increased binding of the Gla-domain to phospholipid membranes and with increased catalytic and anticoagulant effect was highly anticoagulant [25]. Clotting experiments in rat plasma showed distinct dose dependent anticoagulant effect, especially with the mutant QGNSEDY-hAPC: B148 while WT hAPC showed almost no anticoagulant effect. The antithrombotic effect of the different variants of hAPC was then tested in a rat model of deep arterial injury. None of the APC variants had significant antithrombotic effects [25]. However, human PS was not combined with the APC in the experiments, which could explain the poor effects. This hypothesis gained support from additional evaluation of the anticoagulant effect of the hAPC variants *in vitro *using rat plasma, which demonstrated hPS to potentiate the anticoagulant activity of the two hAPC variants. This observation formed the basis for the present study, in which we explore the antithrombotic and anticoagulant effects of co-administration of hAPC and hPS using our arterial thrombosis model in the rat.

## Materials and methods

### Plasma Purified Proteins

Human APC was obtained from Chromogenix (Mölndal, Sweden). Human protein S was purified as earlier described [31]. Bovine protein C was purified as described [32]. It was activated as previously described in detail [16]. Bovine protein S was purified as described [33], with modifications as in [31].

### Recombinant Proteins

The QGNSEDY-hAPC carrying the Gla-domain mutations H10Q/S11G/S12N/D23S/Q32E/N33D/H44Y was prepared as described [28]. The hAPC: B148 mutant was constructed as described earlier [30]. In brief, the 148 loop in hAPC, GWGYHSSREKEAKRN (the underlined amino acids correspond to positions 303–310), was changed to the four residues shorter corresponding loop in bAPC (GWGYRDETKRN). The QGNSEDY- hAPC: B148 mutant was prepared as described [25]. Recombinant hPS was produced as previously described [34].

### *In vitro *anticoagulant analyses

The *in vitro *anticoagulant effects of bAPC, hAPC, and the recombinant hAPC variants with or without protein S (as indicated in the figures) were investigated in rat plasma and in human plasma. Rat plasma was acquired from male Sprague-Dawley albino rats, and the plasma was handled in the same way as in the *in vivo *experiments (see below). Human citrated plasma was acquired from a mixture of plasma from 30–40 volunteers without known coagulation defects. In the APTT system, 50 μl of plasma with or without added bPS or hPS 10 μg/ml (concentration in plasma) were mixed with 50 μl Platelin LS (Biomerieux, Marcy l'Etoile, France) and incubated for 180 seconds at a temperature of 37°C before initiation of coagulation by adding 50 μl of CaCl_2 _30 mM with different concentrations of the APC variants (0, 5, 10, 20, 40, 80, 160 and 320 nM). The experiment was performed three times in duplicate and the mean values of each pair of analyses are presented. In the prothrombin time (PT), 50 μl plasma, with or without added bPS or hPS 10 μg/ml was incubated for 180 seconds at a temperature of 37°C before activation of coagulation by addition of 100 μl tissues factor reagent (Simplastin Excel from Biomerieux), diluted 1:50 in 1 × TBS+0.1% BSA+10 mM Ca^2+ ^(1 × TBS = 50 mM Tris-HCl, 0.15 M NaCl, pH 7.5), containing increasing concentrations of APC variants (0, 5, 10, 20, 40, 80, 160 and 320 nM). The experiment was performed twice and the assays were made in duplicate in one of the experiments and once in the other. The mean values of the double test and the single values of the other experiment are presented. The clotting times were measured up to 200 seconds using an Amelung KC 10 instrument (Amelung Heinrich, Lemgo, Germany).

### APC inhibition in plasma

The chromogenic substrate S-2366 (kindly provided by Chromogenix, Milano, Italy) was used to study the kinetics of inhibition of the APC-variants in rat plasma at room temperature. The concentration of added APC to rat plasma was 50 nM and at different time points, the remaining APC activity was measured with S-2366 hydrolysis at 405 nm in an Automated Microplate Reader (model EL 808, Bio-Tek Instruments, Winooski, VT, U.S.A.).

### Arterial thrombosis model

Sixty Sprague-Dawley male albino rats with a mean body weight of 263 grams (range 226–312 grams) were used. The Research Ethics Committee at Lund University approved the experiments. The experimental model of deep arterial injury was performed exactly as previously described [19,25]. In summary, the right femoral vein was cannulated for infusion of randomized substances and blood sampling. After clamping the left common carotid artery, a longitudinal incision exposed the inner vessel layer. A five millimeter endarterectomy was performed by microsurgical technique and deep vessel layers were exposed. The arteriotomy was closed with micro sutures. The clamps were removed and the vessel was reperfused. All blood emerging from the suture line was collected by preweighed cylindrical swabs to calculate the total blood loss from the arteriotomy. Vessel patency was evaluated 31 minutes after reperfusion with the standard microvascular empty-refill test and the vessel was classed as open or occluded. One surgeon (K.M.) performed all operations. The mean total time for the experiments was 92 minutes (range 84–103).

### Experimental Protocol

In a blind randomized study, 60 rats were divided into 5 groups that were treated either with recombinant hPS 0.6 mg/kg (n = 10), WT hAPC 0.8 mg/kg + hPS 0.6 mg/kg (n = 10), QGNSEDY-hAPC 0.8 mg/kg + hPS 0.6 mg/kg (n = 10), QGNSEDY-hAPC: B148 0.8 mg/kg + hPS 0.6 mg/kg (n = 10) or vehicle only (n = 20). The randomized substances were stored in 1.5 ml coded Eppendorf plastic tubes (Sarstedt, Numbrecht, Germany) at -78°C and thawed to room temperature immediately before use. The substances were delivered as intravenous weight-adjusted bolus injections after completing the vascular injury, one minute before reperfusion. The infusion volume was 0.21 ml per 100-gram animal weight in all groups. The vehicle was 20 mM Tris-HCl, 0.15 M NaCl, pH 7.5, containing 0.2% BSA.

### *Ex vivo *coagulation analyses

Blood samples (0.9 ml) were drawn from the femoral vein of the rats 1 minute before injection of the substance, then 2 and 30 minutes after reperfusion. Before each sample was collected, 0.5 ml of blood was drawn into a separate syringe and wasted. After every 0.9-ml sample was collected, 0.5 ml of saline was injected with another syringe to clear the catheter from blood. The samples were collected with a 2-ml plastic syringe (Plastipak; Becton Dickinson AB, Hägersten, Stockholm, Sweden) prefilled with 0.1 ml of 3.8% 0.13 M sodium citrate. The samples were immediately transferred to 1.5-ml Eppendorf plastic tubes and centrifuged at 14 000 rpm for 3 minutes (Mikro 12/24; Hettich Zentrifugen, Tuttlingen, Germany). The plasma was separated with a pipette and put in two Eppendorf tubes with 300 μl of plasma in each. The tubes were put in a box containing pellets of carbon acid ice. After completion of the daily experiments, the plasma samples were transferred to a freezer maintaining a temperature of -78°C. The APTT and PT assays were done as described above with the exception that the Platelin LS was diluted 1:2 in TBS+ 0.1% BSA.

### Statistical Methods

Patency rates were compared using Fisher's exact test and bleeding weights with the Mann-Whitney test. Statistics were calculated using StatXact 4.0.1 software (Cytel Software Corp., Cambridge, Mass., U.S.A.). Two-sided p-values are presented and p-values <0.05 were considered statistically significant. No correction was made for multiple testing.

## Results

### Anticoagulant effects of human APC variants in rat plasma

**Figure 1:**
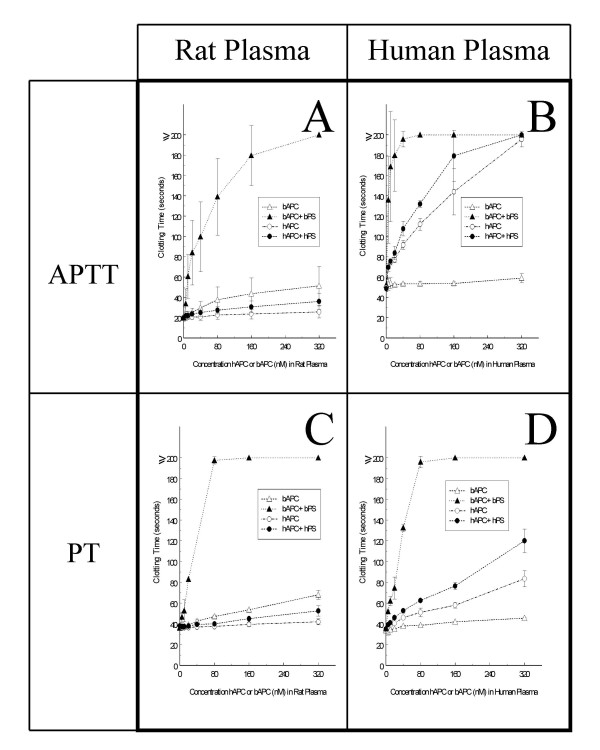
***In Vitro *anticoagulant response in rat plasma produced by plasma purified proteins**. Increasing concentrations of bAPC or hAPC, with or without PS (10 μg/ml), in rat (A+C) or human plasma (B+D) measured with either the APTT reagent Platelin LS (A+B) or with the tissue-factor reagent Simplastin Excel (C+D). Mean values and standard deviation (error bars) are presented. The indicated concentrations of APC refer to the concentrations of the proteins in the APC-containing solutions that were used to initiate the clotting reactions, as detailed in the Methods section. The experiments with bAPC in rat plasma, with or without bPS, were presented in one of our previous studies [19] and the same results are presented here again in the same figure as the experiments with hAPC, with or without hPS, in both plasma types to make comparisons possible.

**Figure 2:**
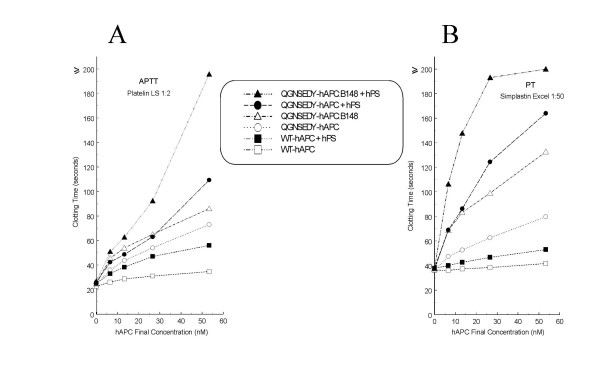
***In vitro *anticoagulant response in rat plasma to added APC variants**. Different concentrations of WT hAPC, or the two mutants of hAPC, with or without recombinant hPS (10 μg/ml) were added in coagulation tests using either the APTT reagent Platelin LS (A) or the tissue-factor reagent Simplastin Excel (B). Note that the APTT reagent was diluted 1/2 to make the assay more sensitive. Therefore, it can be concluded that the hAPC variants + hPS have lower anticoagulant potential than the bAPC+bPS combination shown in figure 1, even though the prolongation of clotting times are similar. Each experiment was performed three times with measurements in duplicate. Mean values are presented. The indicated concentrations of hAPC represent the final concentrations. The experiments without hPS were performed and presented in our previous study [25] and the same results are presented here again in conjunction with the tests with hPS, to make comparisons possible.

Plasma-derived human APC, even when combined with human PS, yielded only a very small anticoagulant effect in rat plasma (Fig. 1). Similar results were obtained with recombinant WT APC and PS (Fig. 2). In contrast, in the presence of hPS, both QGNSEDY-hAPC and QGNSEDY-hAPC:B148 plus hPS approximately doubled the clotting time in the whole dose range tested (Fig 2).

### Rate of inhibition of APC variants in rat plasma

**Figure 3:**
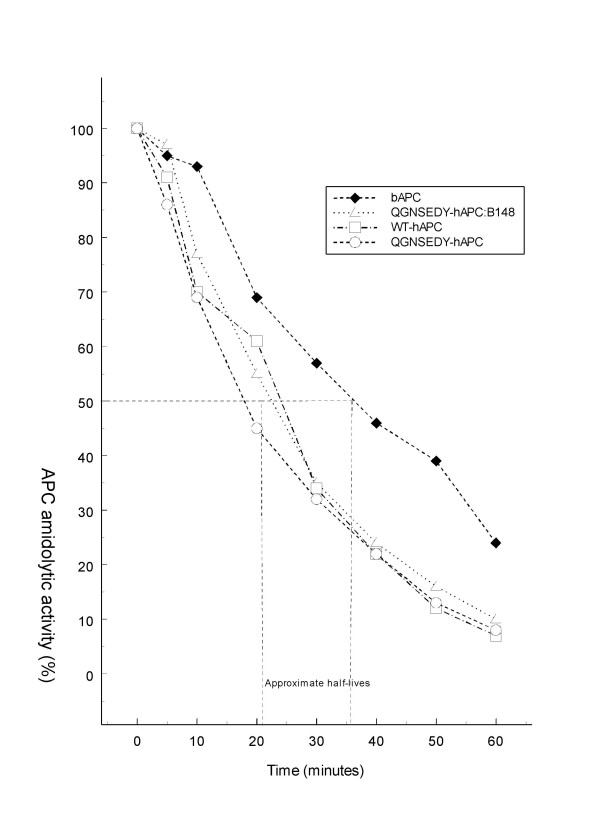
**Inactivation of APC in rat plasma**. The different APC variants were added to rat plasma and at various time points; the remaining amidolytic activity was measured at 405 nm using the synthetic substrate S-2366. The absorbance reading of plain rat plasma was subtracted from the mean of two absorbance readings at each time point and the difference was used for presentation. The three recombinant hAPC variants were tested in one experiment and bAPC was tested in another experiment and the results were combined into one figure.

The APC variants were mixed with rat plasma and the rate of inhibition of enzymatic activity measured (Fig. 3). The recombinant hAPC variants showed similar t_1/2_, around 20 minutes. Bovine APC, used as control, is inhibited at a slower rate with t_1/2_of 35 minutes.

### Antithrombotic effect of hAPC variants?

**Figure 4:**
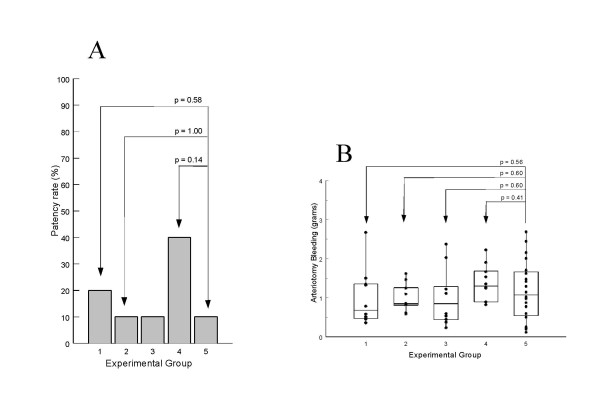
**Effects of WT hAPC and the two mutants of hAPC combined with hPS in the rat model of arterial thrombosis**. Experimental group numbers in A and B refer to the following proteins: 1 = hPS, 2 = WT-hAPC + hPS, 3 = QGNSEDY-hAPC + hPS, 4 = QGNSEDY-hAPC:B148 + hPS, 5 = Vehicle. A. Antithrombotic effect, expressed as the percentage of patent vessels. None of the hAPC variants combined with hPS produced any significant antithrombotic effect, although QNSEDY-hAPC:B148+hPS resulted in an increased patency-rate compared to controls with p = 0.14 (n = 10 in all treatment groups, n = 20 in the control group). B. Arteriotomy bleeding (n = 10 in all treatment groups, n = 20 in the control group). All observations are plotted as well as the 25^th^, 50^th ^(medians) and 75^th ^percentiles (within boxes). There were no statistical significant differences between the groups.

In the groups with WT hAPC+hPS or QGNSEDY-hAPC+hPS there was only one vessel open in each group. In the group with QGNSEDY-hAPC:B148+hPS, there was a trend towards antithrombotic effect as the patency rate was 4/10 (Fig 4A), but the difference to the control group was not statistically significant (p = 0.14). The administration of hPS alone did not give any antithrombotic effect, the patency rate (2/10 vessels) being similar to that of the control group (2/20).

### Arteriotomy bleeding

Administration of the different hAPC variants did not increase the arteriotomy bleeding compared to controls (Fig 4B). The cumulative arteriotomy bleeding during 31 minutes of observation was 1.1 grams (median) in the control group and 0.68 to 1.3 grams (medians) in the treatment groups. There was no statistically significant difference between the groups.

### *Ex vivo *coagulation analyses

**Figure 5:**
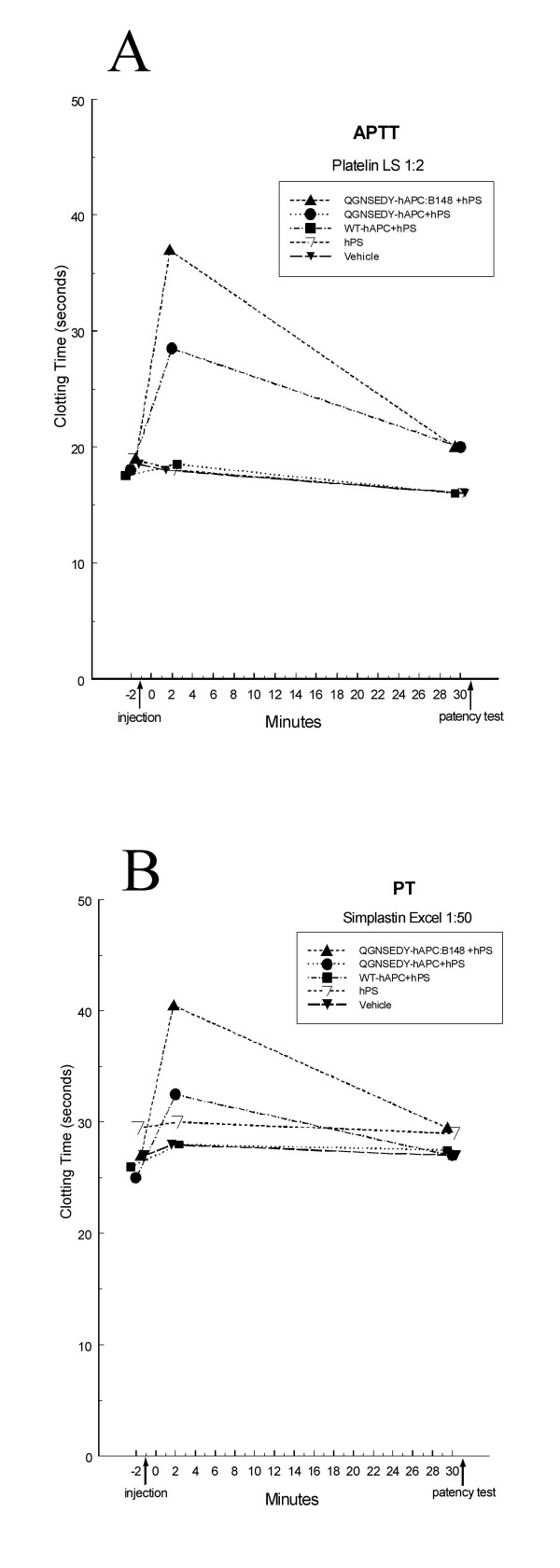
***Ex vivo *anticoagulant response in rat plasma**. The coagulation times were measured with either the APTT reagent Platelin LS (A) or with the tissue-factor reagent Simplastin Excel (B). Baseline values were obtained at -2 minutes. The randomized substances were injected at -1 minutes, followed by clamp release at 0 minutes (n = 10 in all treatment groups, n = 20 in the control group). The medians are shown.

Administration of WT hAPC+hPS or hPS alone only slightly increased the clotting time (Fig 5A and 5B). Three minutes after injection of QGNSEDY-hAPC+hPS, the APTT and PT times increased 1.6 and 1.3 times, respectively. Corresponding values for QGNSEDY-hAPC:B148+hPS were 1.9 and 1.5 times. At 31 minutes after injection, the clotting times had almost returned to baseline levels in all groups (Fig. 5A and 5B).

## Discussion

In our arterial thrombosis model in the rat, we find no antithrombotic effect of WT hAPC [25] even when we now give WT hAPC together with hPS. This is in contrast to results obtained in other thrombosis models in the rat, where hAPC has been reported to have antithrombotic properties [20-24]. The discrepancy may be due to differences in the type of vascular trauma. Our model includes a crude vascular trauma producing occlusive thrombi, and is quite resistant to antithrombotic intervention. It was developed to mimic conditions encountered in clinical vascular surgery. However, despite being arterial and involving primary hemostasis events, the model is also dependent on activation of blood coagulation and sensitive to anticoagulant substances[19,26,27].

The interaction between APC and PS is species-specific, which makes cross-species studies more difficult. However, it can also be an advantage and help clarify the importance of the cofactor function of PS. Thus, we have found bAPC to be a highly efficient antithrombotic agent in arterial thrombosis models with patency rates reaching 90–100% in both rat and rabbit, but only when bovine PS was given together with the bAPC [17-19]. This agrees with results of clotting assays, in which bAPC is highly anticoagulant in human, rabbit, and rat plasmas in the presence of bovine PS, but not in its absence [35,36]. Human APC has shown anticoagulant effects in bovine, baboon and rabbit plasmas, suggesting that hAPC is able to interact with PS from different origin [33-37]. However, we now find almost no hAPC cofactor effect of hPS in rat plasma.

As hAPC was inactive in our thrombosis model, we were interested in evaluating the antithrombotic effects of two hAPC variants (QGNSEDY-hAPC and QGNSEDY-hAPC: B148) having enhanced anticoagulant activity. These mutants, despite giving an anticoagulant response in rat plasma, did not give a significant antithrombotic effect when given alone in our arterial thrombosis model, i.e. no co-administration of hPS [25]. We have now investigated the possible beneficial effects of co-infusion of the hAPC variants with hPS. The results show that neither form of hAPC produced significant antithrombotic effects and although QGNSEDY-hAPC: B148 increased the vessel patency rate compared to the controls, the difference did not reach significance.

The discrepant findings in the thrombosis model between human and bovine APC are interesting. In coagulation tests performed both in vitro and ex vivo, bAPC plus bPS yielded stronger anticoagulant response [19] than the combination of hAPC variants plus hPS (Fig 2 and 5), which should contribute to the lower anti-thrombotic effects of the hAPC variants. Another factor is the difference in elimination kinetics between the bovine and human APC, the half-life of bAPC in rat plasma being almost twice as long as those of the hAPC variants (Fig. 3). APC is inhibited in plasma mainly by two serpins, α_1_-antitrypsin (α_1_-AT) and protein C inhibitor (PCI) [38,39]. Bovine APC has been shown to be resistant to inhibition by α_1_-AT *in vitro *and the structural requirements for this has been investigated [40]. The low anticoagulant effect of hAPC in rat plasma may also suggest that rat FVa and FVIIIa are poor substrates for hAPC. In this context it is interesting that more than ten-fold higher concentration of hAPC was needed to inactivate rat FVa as compared to human FVa [41].

## Conclusion

In conclusion, in an arterial thrombosis model in the rat we find no significant antithrombotic effects of neither WT-hAPC nor hAPC variants with enhanced anticoagulant activity even when the human APC cofactor PS was co-injected. Our results underline the difficulties in using reagents across the species borders and calls for the creation of corresponding APC variants with the same species background as the animals that are used in the thrombosis model.

## Abbreviations

WT: wild type; APC: activated protein C; PS: protein S; APTT: activated partial thromboplastin time; PT: prothrombin time; TBS: tris-buffered saline; BSA: bovine serum albumin.

## Competing interests

KM and BA report no competing interest. BD is inventor of the APC variants on pending patent applications.

## Authors' contributions

KM performed all surgical procedures, participated in planning of the study, interpreted results and participated in writing of the manuscript; BA participated in the development of the thrombosis model, in the planning of the study, interpretation of results, and in the preparation of the manuscript; BD participated in the planning of the study, provided reagents, performed in vitro assays, interpreted results and participated in the writing of the paper.
